# Modelling arts professionals’ wellbeing and career intentions within the context of COVID-19

**DOI:** 10.1371/journal.pone.0292722

**Published:** 2023-10-25

**Authors:** Neta Spiro, Caitlin Shaughnessy, George Waddell, Rosie Perkins, Aifric Campbell, Aaron Williamon

**Affiliations:** 1 Centre for Performance Science, Royal College of Music, London, United Kingdom; 2 Faculty of Medicine, Imperial College London, London, United Kingdom; 3 Central Faculty, Centre for Languages, Culture and Communication, Imperial College London, London, United Kingdom; Jiangsu University, CHINA

## Abstract

The COVID-19 pandemic had a substantial effect on the creative and cultural industries in the United Kingdom (UK), as seen in our first snapshot of the *HEartS Professional Survey* (April–June 2020, Phase 1, *N* = 358). By analysing data collected one year later (April–May 2021, Phase 2, *N* = 685), the aims of the current study are to trace the contributors to (1) arts professionals’ mental and social wellbeing and (2) their expectations of staying in the arts. Findings show that artists continued to experience challenges in terms of finances, and mental and social wellbeing. Over half of the respondents reported financial hardship (59%), and over two thirds reported being lonelier (64%) and having increased anxiety (71%) than before the pandemic. Hierarchical multiple linear regression models, using the Mental Health Continuum-Short Form, Center for Epidemiologic Studies Depression Scale, Social Connectedness Scale, and Three-Item Loneliness Scale as outcome variables, indicate that perceived financial hardship continued to be associated with higher depression and loneliness scores. As in our first study, more physical activity before lockdown was associated with higher wellbeing and social connectedness scores, and higher self-rated health scores were associated with higher wellbeing and lower depression scores. Similarly, increases in physical activity during lockdown, as well as older age, were still associated with higher wellbeing and social connectedness scores and with lower depression and loneliness scores. An ordinal logistic regression model indicated three contributors to artists’ professional expectations of remaining in the arts: greater proportion of income from the arts pre-pandemic, continued maintenance of skills, and greater proportion of freelance work. The results suggest that the wellbeing patterns observed at the start of the pandemic remained consistent a year on. They point to possible strategies to support wellbeing and underline the importance of finances for expectations of remaining in arts professions.

## Introduction

**Fig 1:**
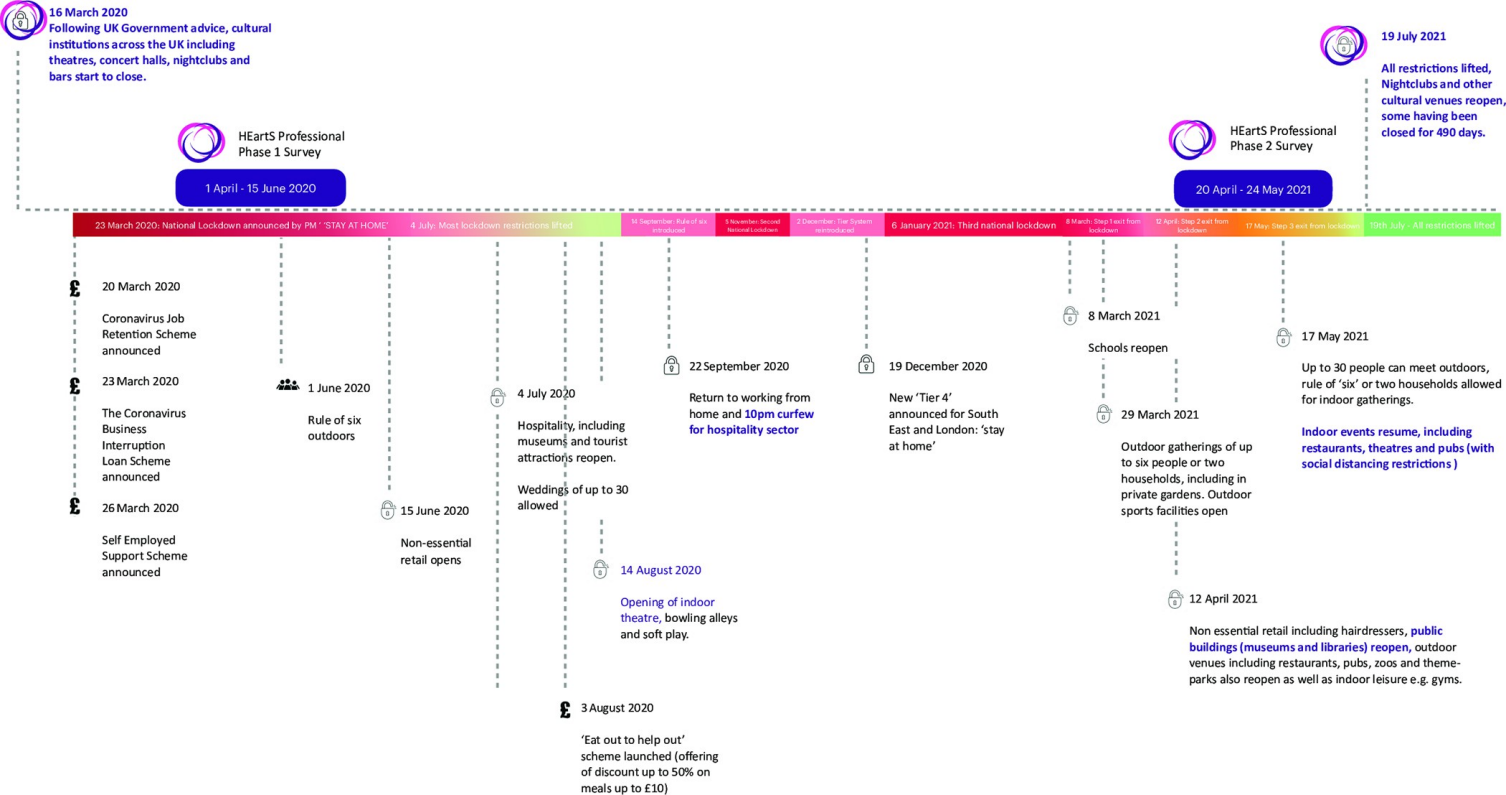
Timelines of data collection in the context of COVID–19 restrictions in the UK (based on [55]).

The COVID-19 pandemic has had profound effects on professional arts workers in the United Kingdom (UK) [1, 2] presenting multiple challenges to their financial, mental, and physical health [2, 3]. Financially, the impact of the pandemic on the arts sector was swift and substantial, as opportunities for performance, creation, and collaboration were suddenly curtailed [1, 2]. Performing venues were shut completely for several months and rehearsals or other forms of artistic engagement, including teaching, were prohibited in person, either stopping entirely or moving online (key dates on which changes to cultural life occurred are summarized in Fig 1 below). For the creative sector in the UK–an industry dominated by freelance workers and short-term projects—this meant that 406,000 jobs were put at risk and some sub-sectors lost more than half of their revenue and workforce [4]. For example, in 2019, the music industry contributed £5.8 billion to the UK economy, and in 2020 this dropped to £3.1 billion: a 46% decrease. Despite the government support schemes, which were announced for businesses and self-employed professionals (including arts professionals), millions of creative freelancers were ineligible to claim and therefore had to turn to alternative forms of work outside the arts industry [5].

A survey study that we carried out early in the COVID-19 pandemic documented that arts professionals had experienced profound effects, but also identified some opportunities and promising links between individual variables and behaviors (such as the role of physical activity) and wellbeing [2]. The purpose of the current study was to continue to trace arts professionals’ work, health, and wellbeing. The aim was to capture another snapshot of work and wellbeing one year into the pandemic and thus trace whether patterns identified in our earlier study were repeated a year later. We also responded to findings in our first study by exploring whether artists saw themselves as remaining in the profession.

### Literature review

In this review of the literature, we draw on industry reports, parliamentary evidence, policy, as well as academic sources to provide the context of the work of arts professionals during the pandemic, their health and wellbeing before and during the pandemic, as well as previously identified associations between wellbeing and the arts.

### Work of arts professionals during the pandemic

Research exploring the impact of the pandemic on arts professionals emerged quickly. For example, organizations that support artists, such as the Musicians’ Union [6] and the Society of Authors [7] carried out surveys to understand the impact on their members. At the same time, academics and third sector organisations tracked the impact on particular groups of arts professionals such as orchestral musicians [8] and visual artists [9]. Across the cultural industries and academia, concerning patterns of lost work, isolation, and health were observed [8–14]. The financial hardships were particularly acute. For example, 38% of professional musicians were not accounted for in the UK governments’ support schemes [15], and revenue from live music within the music industry is not expected to recover fully until 2024 [16]. Policy initiatives provided to mitigate the impact on arts professionals such as the £1.57 billion Cultural Recovery Fund (CRF, [17], launched in July 2020), which provided grants to institutions, and the Self Employment Income Support Scheme (SEISS, [18], launched in March 2020) which provided income support to individuals, only supported some workers with many freelance artists left unable to benefit [19]. At the outset of the pandemic, concerns were raised about the impact upon the diversity of the sector, and as research by the Centre for Cultural Value has since observed, younger artists, people of colour, and disabled workers are now leaving creative occupations at a greater rate than at pre-pandemic levels [3]. Differences in arts specialisms were also seen. In 2020, up to a third of musicians were thinking of leaving the profession [20] and many were questioning their professional identities [8, 21]. Pandemic disruptions were felt more keenly in the performing arts than in sectors that were less reliant on in-person, live activities (e.g., painting), and recovery has been uneven across the sector [13, 14].

### Health and wellbeing of arts professionals

The ongoing impacts of lockdowns and disruption also affected arts professionals’ health and wellbeing. For example, professional musicians have experienced high levels of anxiety and distress [8, 21] and circus performers experienced novel stressors connected with isolation and lockdown [22]. Mental health concerns were also observed among professionals in training. For example, rising trends in anxiety, depression, and suicidality have been identified among performing arts students [23].

The depth of these impacts was anticipated by many researchers as occupational challenges in the arts sector have been long acknowledged. Research previously noted the problems of job insecurity [24–27], challenges in terms of mental wellbeing and depression [28–31], and low social support [32]. Poor physical health has been observed in dancers [24] which is mirrored by potentially harmful perceptions, attitudes, and behaviors toward health and some aspects of fitness in musicians [33, 34]. Rather than the once in a generation ‘force majeure’, it is perhaps more helpful to interpret the impacts of the pandemic as accentuating existing challenges; heightening the precarity of working in the arts and making visible some of the previously ‘invisible’ burdens of creative work, patterns of which have been growing since the financial crisis of 2008 [35].

More broadly, the negative experiences of health and wellbeing by arts professionals follow patterns that are similar to those observed in the wider population during the pandemic. From the start of the pandemic, evidence suggested that quarantine was associated with negative social effects [36], that family or home stressors during the pandemic were associated with poorer mental health outcomes [37], and that worries and experience of adversities that came together during the pandemic—such as financial worries or loss of work—were associated with higher levels of depression and anxiety [38]. A year into the pandemic, a systematic review of risk factors associated with poor mental health [39] found an increased risk of anxiety and depression for particular demographic characteristics including chronic/psychiatric illnesses, gender (female), and age (being under 40). Further distinctions have been identified between the ‘disease anxiety’ caused by COVID-19 that appeared to be more felt by those with physical ill–health and underlying conditions, and ‘consequence anxiety’ of the loss of opportunities and impact on economic prospects that was mostly associated with older adolescents and those with lower incomes [40].

### Positive associations between wellbeing and the arts

Though we began with examples of difficulties experienced by arts workers during and before the pandemic, positive associations between working in the arts and aspects of wellbeing—as well as the more general importance of the arts in economic and health terms for the general population—have long been acknowledged. As discussed by Spiro, Perkins et al. [2], engagement in arts and culture, in addition to being valued in and of itself [41], has been shown to contribute to public health especially in terms of mental and social wellbeing [42, 43]. During COVID-19 it was observed, for example in a study in Spain [44], that the public turned to the arts to support their mood and feelings of companionship. Furthermore, the arts played a role in bringing people together at a time of crisis, as seen in actions of high profiles artists, such as the global *Together at Home* concert, which raised £128 million for COVID-19 relief funds [45].

Work in the arts can also have positive associations for the professionals themselves. For example, a survey study carried out before COVID-19 explored the PERMA profiler (consisting of five elements that are associated with flourishing: Positive Emotion, Engagement, Relationships, Meaning, and Accomplishment) [46] with professional classical musicians [47]. Musicians scored significantly higher than the general population on “Positive Emotion” (which refers to the affective component of feeling well), and on “Relationships” (which refers to the perception of quantity and quality of social connections) [47]. Musicians also scored higher than the general population on “Meaning”, referring to the feeling of belonging or serving something larger than the self [47]. Meaning, in turn, has been associated with better physical health and higher life satisfaction [48, 49]. Alongside these findings, creative careers are also associated with a strong sense of self identity and personal motivations that are emotionally significant, with arts work often viewed as a way of life rather than simply a source of income [50, 51].

Even during the first lockdown, some workers in the arts experienced professional and personal opportunities, suggesting that the pandemic impacts had both positive and negative factors [2, 11]. These included respondents living more healthily, having more time, and experiencing less pressure in their day-to-day and professional lives. They also reported identifying new ways of working online, re-evaluating their life circumstances, and finding new creative opportunities. Moreover, they experienced enhanced social connections through strengthened connections with family, other performing artists, and the local and wider communities. Early career artists also noted that the experiences of lockdown enabled more time to develop artistically and to learn new skills in both digital and creative areas [11].

As the literature highlighted above suggests, arts professionals had a range of both negative and positive financial and wellbeing experiences during the pandemic. The previous literature also indicates that whilst many of these issues were not new, the pandemic magnified concerns and considerations of their implications both on individual and organisational levels. Previous studies had not looked systematically across the cultural sector and over time to explore the finances, support, and mental and social wellbeing of arts professionals in the UK, or their career intentions at this stage in the pandemic.

### Tracing work and wellbeing during the COVID-19 pandemic

Looking across the arts sector, our previous study [2], for which data were collected in April–June 2020, during Lockdown 1.0 of COVID-19 in the UK, indicated a substantial reduction in work and income for professional artists. Over half of the respondents (53%) reported financial hardship, over three quarters (85%) reported increased anxiety, and around two thirds (63%) reported feeling lonelier than before the crisis. Multiple regression analyses, using mental and social wellbeing scales as outcome variables, indicated that self–rated health and physical activity before and during COVID-19 predicted mental and social wellbeing scores. Higher self-rated health was associated with higher wellbeing and lower depression scores (on the Mental Health Continuum-Short Form and Center for Epidemiologic Studies Depression Scale respectively). More physical activity before lockdown was associated with higher wellbeing and social connectedness scores (Social Connectedness Scale), as well as lower loneliness scores (Three-Item Loneliness Scale), and an increase in physical activity during lockdown compared with before was associated with these same outcomes as well as with lower depression scores. The same regressions indicated that perceived financial hardship was associated with lower wellbeing and higher depression and loneliness scores. Several open questions arose from this research including: What would be the ongoing impact on mental and social wellbeing after the first months of dramatic change? Would these disruptions affect arts professionals’ expectations of continuing to work in the arts?

For arts professionals, the intersecting factors of the limitations on work, exposure to COVID–19 through professional activities, and the wider impacts of the pandemic on health and wellbeing suggest that they may have faced particularly challenging times. While research in the sector was quick to respond to the immediate impact of the pandemic, the lack of clarity regarding how arts professionals’ work, wellbeing, and expectations of staying in the arts would be affected by the pandemic and its associated changing restrictions required time-sensitive and consistent tracing as the pandemic developed. Therefore, one year into the COVID-19 pandemic this study investigated the contributors to (1) arts professionals’ mental and social wellbeing and (2) their expectations of staying in the arts through two related research questions:

RQ1. What are the contributors to UK arts professionals’ mental and social wellbeing one year into the COVID-19 pandemic and how do these relate to contributors identified in previous models?

RQ2. What predicts UK artists seeing themselves as having a future working in the arts?

Both research questions were addressed by providing a snapshot collected through an online survey in the UK approximately one year into the COVID-19 pandemic (April–May 2021). The survey explored respondents’ mental and social wellbeing as well as finances, work experiences, health, exercise, and demographic characteristics.

## Method

### Respondents

The survey was open to professionals working in the arts in any capacity in the UK (S1 File, questions 4.1 and 4.2). In this phase 685 respondents completed the survey. About half of the respondents (*n* = 337, 49%) worked in the visual arts (with film/video making/photography being highly represented, *n =* 174). Just under a third worked in the performing arts (*n* = 218, 32%) such as acting, dancing, and musical theatre, and a similar proportion (*n* = 199, 29%) worked in music or sound arts (with classical music being highly represented among those working in sound arts, *n* = 112, 56%). 11% worked in literature (*n* = 74). Respondents could select more than one area of work. Demographic characteristics of the arts professionals based in the UK are summarized in S1 Table.

About two thirds of the respondents identified as female (*n* = 436, 64%). Approximately 70% of the respondents were aged between 26 and 55 (*n* = 499, mean age = 37.74, *SD* = 13.35). The majority of respondents were white (*n* = 606, 89%), about half of the respondents had a tertiary degree (*n =* 399, 58%) and about a quarter had an advanced qualification (e.g., a masters, PhD, DMA, DMus degree, *n* = 182, 27%). London was home to the largest group of respondents (*n =* 211, 31%). Almost a quarter lived with children (*n =* 147, 22%), and under 20% lived alone (*n =* 89, 13%). Around a quarter (*n* = 160, 23%) had a household income of £52,000 or more, although household income spanned the whole range of the scale (£0–£76,000+). On average, our respondents contributed to over half of that income (53%) with the majority of their contributed income coming from work in the arts (mean = 63%). In terms of employed compared to freelance work, just over half (53%) reported earning the most of their income through freelance work with almost three quarters (74%) reporting having at least some freelancing as part of their work portfolio. Most respondents at the time of data collection had not had (or not knowingly had) COVID–19 (*n* = 559, 82%) (S1 Table).

The majority of respondents positively rated their health (*n =* 494, 72% rating it “Very good” or “Good”). The majority did not report chronic health conditions (*n* = 477, 70%). Just under half reported a reduction in physical activity in the last month (*n =* 292, 43%) (S2 Table).

As in our previous article, we examined how similar our sample of respondents is to performing arts professionals in the UK [2]. According to the 2018–2019 Arts Council report on equality and diversity in the institutions they support, 60% identified as female and, out of the workers about whom ethnicity is known, 83% were white [52]. Although the Arts Council report does not cover all workers in the arts and cultural sector, this comparison suggests that our sample is close to the proportions of the sector on at least some demographic characteristics.

For those performing, an overwhelming majority of respondents reported a reduction in working time *performing* (*n* = 174, 90%). Working in other areas was reduced, for example reduction in time spent *teaching/coaching/workshop leading/mentoring* was reported by 57% of respondents (*n* = 98). Looking across all areas of work, 52% of respondents spent less time working than before (Table 3A in S3 Table).

Respondents’ modes of work were decisively online. Looking across all disciplines and work activities, 77% of work was online (alone and with others) and 23% was offline (alone and with others). Skill maintenance and development was still limited, 52% had either not done, or done less *learning/practicing/preparing/reflecting* with 15% seeing no change and 33% doing more. Beyond work, socializing in person was still less than pre–pandemic levels, with 85% seeing fewer people. Conversely, socializing online was still high with 64% seeing more people online (Table 3D in S3 Table).

The demographic characteristics of the survey respondents for phases 1 and 2 were, in general, similar to those from the first snapshot survey collected at the start of the pandemic (Phase 1, [2]) but Phase 2 was more diverse. For example, the sample in Phase 2 included respondents with a wider range of arts professions (with a greater proportion of those who work in the visual arts and literature as well as the original areas of performing arts). The sample in Phase 2 was slightly younger (a mean age of 38.00 compared with 44.08 years in Phase 1), and ethnically a slightly more diverse demographic (with 93% identifying as White in Phase 1 and 89% in Phase 2). A lower proportion reported having an advanced qualification (e.g., masters, PhD, DMA, DMus degree, 51% in Phase 1 compared with 27% in Phase 2). A smaller proportion of respondents lived in London (42% in Phase 1 compared with 31% in Phase 2) and a smaller proportion had a household income of £52,000 or more (36% in Phase 1). Working time, modes of work, and socializing patterns were consistent with our sample in Phase 1.

### Procedure

Data were collected via *HEartS Professional* (see S1 File), a survey of workers in the arts and cultural sectors to determine the impact of the COVID–19 pandemic on their health, wellbeing, and livelihoods. *HEartS Professional* is an adaptation of the *HEartS Survey* which charts the Health, Economic, and Social impacts of the ARTs [53]. *HEartS Professional* was designed as a multi–strategy data collection tool with two main purposes: (1) to chart working patterns, income, sources of support, and indicators of mental and social wellbeing in order to identify trends in the effects of the pandemic; and (2) to explore the individual work and wellbeing experiences of performing arts professionals in their own words. The survey therefore covered six areas: (1) demographics; (2) information on illness or self-isolation related to COVID-19; (3) work profiles and income; (4) changes to work profiles and income as a result of the pandemic, as well as sources of support; (5) open–response questions about work and wellbeing experiences of lockdown including challenges and opportunities; and (6) validated measures of health, wellbeing, and social connectedness [2]. This study reports on areas 1–4 and 6 of *HEartS Professional* in order to chart working patterns, income, sources of support, and indicators of mental and social wellbeing and to identify quantitative trends in the effects of the ongoing pandemic on arts professionals. For discussion of the qualitative findings from the open responses (Area 5), see Shaughnessy et al. [54].

Approval was granted by the Conservatoires UK Research Ethics Committee. Informed consent was obtained at the beginning of the survey. The intention was to recruit as broad a spectrum of people as possible who were over the age of 18 years, working in the arts and cultural sector, and living across the UK. These three factors thus formed our recruitment criteria.

For Phase 2, we recruited respondents through two routes. First, we returned to respondents who had participated in Phase 1 via email. Second, we contacted respondents through the online survey platform Prolific (https://app.prolific.co/). In Phase 1 we had asked respondents to provide their emails if they were interested in participating in future surveys. To ensure anonymity, email addresses were stored separately from the data. Of the 241 respondents who provided email addresses in Phase 1, 176 consented to participate and of these 140 completed the survey. Thus around 36% of respondents from Phase 1 (*N* = 385) completed Phase 2. Put another way, only 16% of respondents in Phase 2 were the same as in Phase 1. We therefore did not compare between the same respondents in the two phases. Respondents recruited through Prolific were paid the equivalent of £8.97 (GBP) per hour on survey completion. The median completion time was 20 minutes. Of the 582 respondents that clicked on the advertisement for the survey through Prolific, 575 completed the consent, and 548 completed the full survey (i.e., 95% of those who clicked on the link for the advertisement. The Phase 2 survey was open from 20^th^ of April to 24^th^ of May 2021. As illustrated in the timeline (Fig 1), Phase 1 of data collection occurred early on during the pandemic and Phase 2 (the data reported on here) occurred while cultural venues began reopening.

Taking respondents who reached the survey from both routes together, of the 751 people who completed the consent section of the survey, 688 reached the final question, which gives a completion rate of 92%. All the closed questions (i.e., all those in sections 1–4 and 6) were compulsory. We did however exclude cases from the dataset if there was evidence of response bias such as straightlining (*n* = 1) or extreme responses (*n* = 2). This left us with a sample of 685 respondents. Copies of the dataset, with some data redacted to follow Dryad’s protocols, are publicly available (https://doi.org/10.5061/dryad.6t1g1jx2v).

### Outcome measures

As described by Spiro, Perkins et al. [2], in this study we are led by the view that both positive- and ill-health contribute to how we experience our mental and social wellbeing [56]. We therefore used one outcome measure each for a positive view as well as a symptom-led view of mental and social wellbeing. More specifically, for mental wellbeing we focused on mental health and depression, and for social wellbeing we focused on social connectedness and loneliness. For mental wellbeing we used the 14-item Mental Health Continuum-Short Form (MHC-SF) [57, 58]. The MHC-SF measures hedonic dimensions (3 items) and eudaimonic dimensions (11 items). Each of the 14 items is rated on a 6–point scale (0 “never”, 1 “once or twice”, 2 “about once a week”, 3 “2 or 3 times a week”, 4 “almost every day”, 5 “every day”) generating a continuous score ranging from 0–70. In addition, a categorical variable can be derived denoting: “flourishing mental health” (for respondents experiencing at least one of the three hedonic dimensions and at least six of the eleven eudaimonic dimensions “every day” or “almost every day”), “languishing mental health” (for respondents experiencing at least one of the three hedonic dimensions and at least six of the eleven eudemonic dimensions “never” or “once or twice” in the past month), and “moderate mental health” (for respondents in between the two previous categories). For depression, we used the eight-item Center for Epidemiologic Studies Depression Scale (CES-D). Each of the eight items has Yes or No response options and the number of depressive symptoms present is summed, generating a score ranging from 0–8. A score of three symptoms or more (out of a possible eight) has been commonly used for identifying cases of depression [59]. For social connectedness we used the 15-item Social Connectedness Scale [60], where a higher score indicates more connectedness to others. For loneliness, we used the Three-Item Loneliness Scale [61, 62], which identifies those scoring six or higher out of a possible nine as lonely.

### Analysis

Data were analyzed using descriptive statistics to provide an overview of general patterns of work, income, perceived changes in anxiety and loneliness, and support sought. We ran four separate hierarchical multiple linear regression models using jamovi (2.2.5.0; The jamovi project, 2021) to explore the relationship between COVID-19-related, demographic, and arts work variables and the levels of mental wellbeing, depression (following [63]), social connectedness, and loneliness (following [64, 65]).

To model mental and social wellbeing (Research Question 1), four regressions were run (one for each outcome variable, each with two models):

- Model 1 was adjusted for three pandemic–specific variables: physical activity during the pandemic (Lockdown exercise), perceptions of financial hardship (Financial hardship), and changes in socializing with others, both online and in–person (Socializing change) (S5 Table details these variables).
- Model 2 adjusted for covariates related to demographic and work characteristics. Variables associated with demographic factors and arts work were: gender, age, ethnicity, living status (Living alone), self–rated health (Health), exercise habits prior to COVID–19 (Pre–COVID–19 exercise), educational attainment (Ed. Attainment), art specialism, household income (Household income, which includes all earnings including for example, from pensions), percentage of time spent freelancing (% freelance), individual contribution to household income (% Cont. income), and the percentage of one’s individual contribution to household income generated from arts work specifically (% Cont. art).

Ordinary least squares regression assumptions were checked: the assumption of normality of residuals was tested using kernel density plots, standardized normal probability (P–P) plots, and quantile (Q–Q) plots, while homoscedasticity of residuals was established by plotting residuals versus predicted values. No issues of multicollinearity were identified and no outliers were identified for removal.

To explore contributors to performing artists seeing themselves as having a future in the arts (Research Question 2), one regression was run. As an ordinal outcome variable, four categories were defined: respondents who anticipated (1) definite immediate work in the arts (full or part-time), (2) work returning in the future (full or part–time), (3) possible work in the arts (“maybe”; full or part–time), or (4) leaving the arts. We used ordinal logistic regression analyses to investigate which predictors were associated with these findings:

- Model 1: ‘Health and wellbeing’ examined the four outcome variables above (wellbeing, depression, social connectedness, and loneliness), physical baseline, connection to others in the arts, change of socialization, impact of long-term COVID symptoms, and overall self-reported Health.
- Model 2: ‘Finances and employment’ examined financial hardship, income, percentage of household income, freelance income, and income from arts, whether professional skills were being maintained and anticipated maintenance in the future, and whether income lost to the pandemic was expected to be recuperated.
- Model 3: ‘Demographics’ included age, gender, ethnicity, and educational attainment.
- Model 4: ‘Arts specialism’ included whether they worked within the main artistic sectors examined (each as a binary variable), length of time worked in the arts, and whether they worked in performing/teaching/conducting.

## Results

### Financial hardship and support

**Fig 2:**
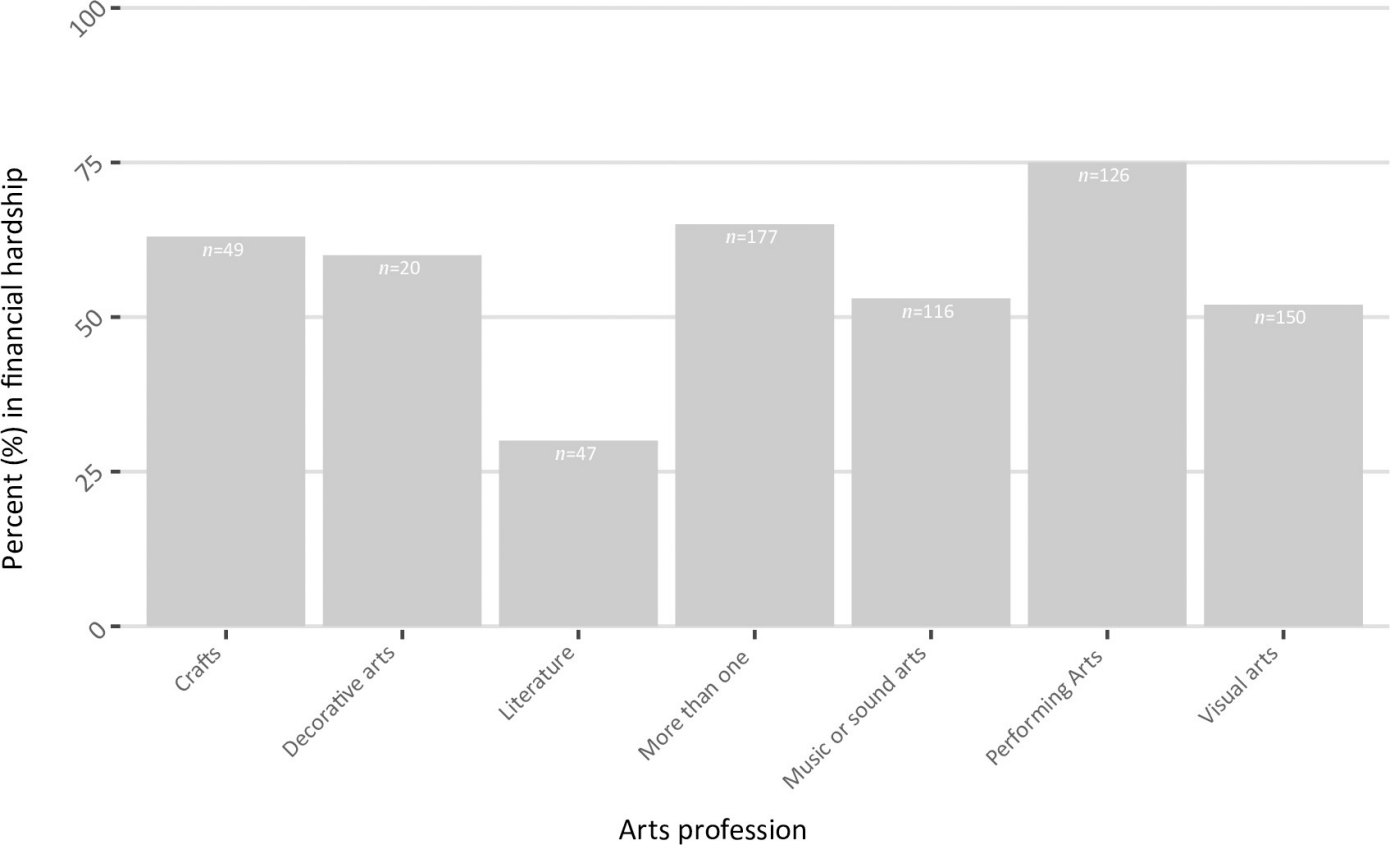
Distribution across the different arts professions reporting that they consider themselves to be in financial hardship as a result of the pandemic (*n =* 405).

Across the cohort, 59% considered themselves to be in financial hardship (see Table 3F in S3 Table). As Fig 2 illustrates, we see rather different proportions reporting financial hardship across the different arts areas. Three quarters (*n* = 94, 75%) of the 126 respondents working purely in the performing arts saw themselves in financial hardship, while only about a third (*n* = 14, 30%) of the 47 people working in literature saw themselves in financial hardship as a result of the pandemic. In between, just over half (*n* = 61, 53%) of the 116 people working in music and sound arts saw themselves in financial hardship as a result of the pandemic.

Of the 405 respondents who reported experiencing financial hardship, 232 (57%) sought support on finances, and about the same number (*n* = 228, 56%) did so for health and wellbeing (Table 3H in S3 Table). The most popular sources of support were family/friends on finances (66%) and on health (71%). Just under half (46%) turned to government agencies on finances and to health professionals (44%) on health and wellbeing.

### Mental and social wellbeing and their associated variables

**Fig 3:**
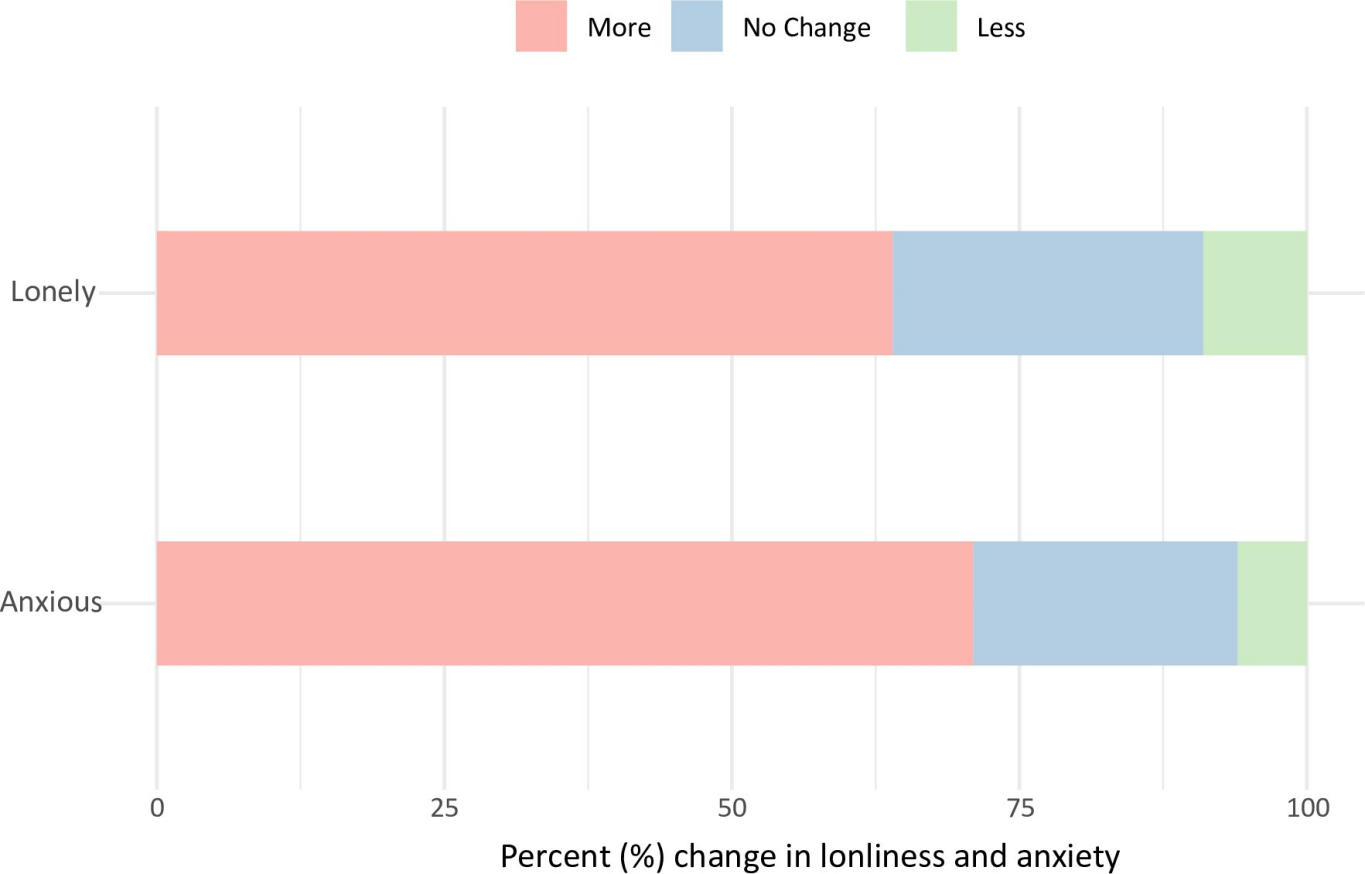
Proportion of change in loneliness and anxiety for all respondents (*n* = 685).

We explored feelings of change in loneliness and anxiety by asking respondents to respond to two 1–item questions “In the last month, how has the public health situation affected how [lonely/anxious] you feel?” on a 7–point scale (Much more lonely/anxious–Much less lonely/anxious), here grouped into three categories (More, No change, Less). The proportion of respondents feeling lonelier in the last month was 64%, (*n* = 436) and the proportion of people feeling more anxious was 71% (*n* = 487) (Fig 3. See also Table 3G in S3 Table).

Using the 14-item MHC-SF scale, over half of respondents reported “moderate” levels of wellbeing (56%). A quarter scored as “flourishing” (25%) and almost a fifth scored as “languishing” (19%). 73% reported three or more depressive symptoms on the 8–item CES–D scale, and hence could be described as depressed ([59]; S4 Table); the mean score was 4.27 (out of 8, *SD* = 2.54). The mean score for social connectedness was 39.46 (*SD* = 15.38) on the 15-item Social Connectedness Scale-Revised which has a range of 0–75. Nearly half (53%, *n* = 362) of the respondents scored 6 or higher out of a possible 9 on the Three-Item Loneliness Scale [61, 62] and were therefore classed as lonely [66] with a mean score of 5.56 (*SD =* 1.76).

### Modelling arts professionals’ mental and social wellbeing

As described above, four regression models were conducted to examine which factors were predictive of outcome scores. Model 1, adjusting for the COVID–19 related factors only, predicted between 4% and 5% of the variance in the outcomes (all with a significance of p < .001, Tables 5A–5D in S5 Table). The fully–adjusted model, which included also demographic and work characteristics, (Model 2) explained 11% of the variance in wellbeing (MHC-SF, adjusted *R*^*2*^ = .114, *F*_15,575_ = 6.08, *p* < .001), 14% of the variance in depression (CES–D, adjusted *R*^*2*^ = .139, *F*_15,575_ = 7.35, *p* < .001), 11% of the variance in social connectedness (Social connectedness scale, adjusted *R*^2^ = .108, *F*_15,575_ = 5.75, *p* < .001), and 9% of the variance in loneliness (Three-Item Loneliness Scale, adjusted *R*^2^ = .092, *F*_15,575_ = 5.34, *p* < .001).

Self–rated health was predictive of all four outcomes variables: higher wellbeing (*B* = 3.31, *p <* .001) and social connectedness scores (*B* = 3.23, *p <* .001), and lower depression (*B* = -0.76, *p <* .001) and loneliness scores (*B* = -0.28, *p <* .001). Physical activity prior to COVID-19 (Pre-COVID-19 exercise) was associated with higher wellbeing scores (*B* = 0.42, *p =* .012) and higher social connectedness scores (*B* = 0.41, *p =* .033). Physical activity during the previous month was associated with higher wellbeing scores (*B* = 0.96, *p* = .003) and social connectedness scores (*B* = .95, *p* = .010), and with lower depression (*B* = -0.17, *p* = .005) and loneliness scores (*B* = -0.13, *p =* .001).

Higher scores for socialization with others were positively associated with wellbeing (*B* = 0.89, *p* = .007) and social connectedness (*B* = 1.45, *p* = < .001). Perceived financial hardship was associated with higher depression and loneliness scores (*B* = 0.63, *p =* .003; *B =* 0.51, *p* < .001).

Age was associated with all four outcomes measures. Older age was positively associated with increased wellbeing (*B* = 0.12, *p* = .007) and social connectedness (*B* = 0.13, *p =* .009), and negatively associated with depression (*B* = -0.03, *p* < .001) and loneliness (*B* = -0.03, *p* < .001). Gender emerged as significantly associated with depression, where men reported lower depression scores compared with women and non-binary respondents (*B* = -0.51, *p* = .015).

### Anticipating a future working in the arts

Turning to our second research question, in response to the question “Do you anticipate a future for yourself working in the arts and cultural sectors?” a very small proportion responded “No” (3%, S6 Table). A majority selected one of the options that included “Yes, purely in the arts” or “Yes, in the arts alongside non–arts work” (*n =* 479, 70%). Almost 30% chose options that included “not at the moment” or “maybe”. These were divided across 13% who chose “Yes, but not at the moment” whether purely in the arts or alongside other work (*n* = 90) and 14% who chose “maybe”, whether purely in the arts or alongside other work (*n* = 95). In total, 332 responded that they anticipated a future for themselves in the arts “alongside other work” whether now or “not at the moment”; this equated to 50% of those anticipating staying in the arts.

One regression analysis was conducted to examine the contributors to performing artists seeing themselves as having a future in the arts. Model 1 (Health and wellbeing) was significant (χ^2^_(9)_ = 33.06, *ΔR*^*2*^_*McF*_ = .031, *p* < .001), accounting for 3% of variance in the outcome variable in which higher scores on wellbeing (*odds ratio* = 1.02, *p* < .05, *95% CI*_*odds*_ = 1.00–1.04) and connections to others in the arts (*odds ratio* = 1.18, *p* < .05, *95% CI*_*odds*_ = 1.02–1.36) predicted higher confidence in future of working in the arts (S7 Table). However, introducing Model 2 (Finances and employment), which was a significant contributor (*χ^2^*_*(8)*_ = 41.56, *ΔR*^*2*^_*McF*_ = .039, *p* < .001), overrode those associations, accounting for a total 7% of variance in the outcome variable, with the primary drivers of higher future confidence being the degree to which individuals were already seeing a higher percentage of income via the arts before the pandemic (*odds ratio* = 1.01, *p* < .005, *95% CI*_*odds*_ = 1.00–1.01), how much of their work constituted freelance activity (*odds ratio* = 1.01, *p* < .05, *95% CI*_*odds*_ = 1.00–1.01), and whether they had been maintaining their skills (*odds ratio* = 1.32, *p* < .001, *95% CI*_*odds*_ = 1.13–1.53). Models 3 (Demographics) and 4 (Arts specialism) did not significantly contribute to the overall model (*ΔR*^*2*^_*McF*_ = .004 –.006, *p* > .05), even when entered ahead of Models 1 and 2.

## Discussion

The results of the regression analyses regarding mental and social wellbeing outcomes suggest that physical activity and health factors (better self-rated health, more physical activity pre-COVID-19, and physical activity during the pandemic) predicted better scores on at least one mental wellbeing and at least one social wellbeing outcome in April–May 2021. We saw that when modelling mental and social wellbeing a year apart, with reference to Spiro, Perkins et al. [2], the models were broadly stable. Physical activity continued to be associated with positive outcomes across all models; however, there was an increased role of socialization and self-rated health in comparison with the beginning of the pandemic in April–June 2020 (Phase 1). Older age continued to be associated with higher wellbeing and social connectedness scores and with lower depression and loneliness scores. Similarly, gender continued to be associated with depression. The results of the second set of regression analyses, carried out only in Phase 2, highlight the importance of financial considerations for anticipating whether professionals will remain working in the arts. Issues of wellbeing and feelings of connections to other arts professionals are present but are overridden by financial and work considerations: pre-pandemic proportion of income from the arts, proportion of freelance work, and skill maintenance.

In our discussion of the results from Phase 1 [2], we commented that our findings of the connections between financial hardship and poor mental health in the arts were in line with research preceding the pandemic in the general population [67], as were associations between gender and depression [68]. We highlighted then that future research was needed to trace whether these associations remain for the creative workforce. With this study, we see that they continued to do so. In terms of the factors associated with mental and social wellbeing outcomes, it is striking that the importance of *physical activity* and *self-rated health* remained. This further supports previous research that has suggested the importance of physical health in the performing arts [24, 34, 69] and during COVID-19 [70]. Although our data do not provide information about causal relationships, the findings highlight the persistence of a relationships between exercise and positive mental and social wellbeing in the arts sector.

Looking across the cohort more broadly, financial hardship has remained a concern, increasing from 53% in Phase 1 to 59% in Phase 2. Those in financial hardship were still seeking support in similar proportions. In Phase 1, 61% sought support on finances, only 45% on health and wellbeing. In Phase 2, 57% sought support on finances, with a slight increase in those seeking support for health and wellbeing (56%). The results also highlight continued patterns in loneliness (63% in Phase 1, 64% in Phase 2), with a slight drop in those experiencing greater anxiety (85% in Phase 1, 71% in Phase 2).

The consistency of the findings across the two phases may not be surprising given that, in reality, little had changed for many arts professionals between the two time points. Furlough schemes were still in operation, many venues were subject to restrictions, and self-isolation was still required particularly for younger professionals who had not yet been vaccinated. However, the consistency of the findings among a largely new sample does support the validity and reliability of the initial models. The fact that Phase 2 of data collection included a more diverse sample both demographically (in particular, concerning education, geographical distribution, and ethnicity) and in terms of arts area also broadens the generalizability of the models.

### Contributors to mental and social wellbeing

While we found that financial impacts across different arts areas were highly variable, the connections with mental and social wellbeing appears to be more consistent across the groups that we surveyed both across and within the two phases. This perhaps reflects the multiplicity but also shared experiences of arts professionals during the pandemic. These could include both problematic concerns, such as anxieties about the future of the cultural industries, and positive outcomes, such as the role of exercise in wellbeing and the flexible adaptability of those with portfolio careers (see also [11]).

Despite the consistency of the models in overall mental and social wellbeing, the situations in 2020 and 2021 were not identical. For example, we see an increased role of general health. COVID-19 is known particularly to affect people with underlying health conditions, so this finding may be connected to the mental health impact of concern over contracting COVID-19 and the heightened precautions that people with underlying conditions might have taken, having even less social contact during the previous year than others. We also see an increased role of frequency of socializing; people who had socialized more scored better on mental wellbeing and social connection, as has been seen in the general population [71]. Alongside the findings on age–with older people scoring lower on the depression outcome–these results highlight the potentially greater detrimental impacts of restrictions on younger arts professionals. This may be connected to the observations that younger people tend to have a more diverse range of social relationships [72] and may have been more affected by the limitations on socializing within these networks [73].

### Career intentions and support

In both phases, we saw that over half of respondents considered themselves to be in financial hardship as a result of the pandemic. In Phase 2, with our larger sample, we were able to break down the responses by arts area. Although the numbers in some groups are low and we exercise caution in interpretation, we see that there are differences in proportions, with three-quarters of the respondents working purely in the performing arts seeing themselves in financial hardship, just over half of respondents working in music and sound arts, and under a third of respondents working in literature. There may be several contributing factors to these results. As already observed in discussion of Phase 1 [2], the context and nature of the work itself may play a key role; it is particularly difficult to earn when a person’s work relies on live, in-person performance while perhaps the craft of writing may continue more easily under social restrictions or in isolation, especially if the end result is printed text rather than a script or score for a live, in-person production. Another factor may be the structure of work and income; many authors had to have other work alongside their arts careers before the pandemic, so changes to their writing income may have had less of a direct effect. For the professions in between, like those in music and sound arts, people who had been predominantly earning through teaching perhaps faced a different challenge from those who had been predominantly earning through performance. Finally, representation may be important. Some arts professionals may have access to professional bodies that are more able to address their conditions directly, as is the case for some musicians. Others, such as puppeteers, may have less access to professional bodies.

It is notable that half of respondents (50%) responded that they intended to continue working in the arts alongside other work. Whether through necessity or through design, arts professionals are anticipating portfolio careers that include non-arts work. Although data on the proportion of secondary work in the cultural sector are limited, the proportion is in line with Throsby and Zednik’s [74] estimations of the proportion of second jobs in the Australian cultural sector but is far higher than the proportion of cultural workers reporting having second jobs in the UK (8%) ([75], see also [76]). Given that our results suggest that artists’ proportion of income from their arts work predicts their anticipation of staying in the arts, the high proportion of expected portfolio careers including non-arts work raises questions about sustainability. Questions remain in terms of identifying what proportion of this is a continuation of pre-pandemic patterns as opposed to what proportion is temporary and directly connected to the pandemic. Moreover, if the anticipated rise in portfolio careers comes to pass, examining the relationship between the arts work and the other work, and the effect that portfolio careers have on health, wellbeing, and sustainability for arts professionals will be more urgent than ever. These findings emphasize the central role of financial stability as the deciding factor for staying in the arts, overriding other predictors.

A further area that saw some change was respondents’ patterns of seeking support. Unlike in Phase 1, in Phase 2, over half of those questioned sought help about health and wellbeing and finances. This may be connected to greater need for or awareness of sources of support. However, the most common sources of support were the informal routes of family and friends. Beyond family and friends, the other avenues for seeking support were more diverse, although government schemes were common avenues for finances and health professionals were most commonly approached for health support. Early in the pandemic in the UK, organizations such as performing arts charities and trade unions made information about support available [77–80]. This suggests that awareness was growing regarding sources of support, but more is needed to open up these avenues across the sector.

### Implications, limitations, and future directions

In light of the known strains of freelance and creative work on the wellbeing of arts professionals before the pandemic [50, 81, 82], the patterns identified here present a worrying picture of the state of the workforce, and the importance of support as working patterns resume. These results highlight patterns that have been seen consistently in research across the sector. Notably, the importance of finances for professional and personal wellbeing, and the extent to which these have been negatively affected by the pandemic, has also been published in cross-sector industry reports by the Centre for Cultural Value [83] and the Creative Industries Policy and Evidence Centre (Creative PEC) [14], as well as in academic research [84, 85]. This study focused on arts professionals which, as a group, had specific experiences of lockdown and the pandemic. However, it is striking that the factors associated with mental and social wellbeing, particularly age and gender, are replicated in research concerning a wider population [86, 87]. Taken together with the observations about the detrimental role of financial hardship in maintaining wellbeing, these results are in line with the multifaceted models of contributors to mental and social wellbeing outcomes [40].

In our previous study, we commented that there is, of course, much variety within the arts sector and many professionals’ situations are specific to them or a subgroup of professionals like them. Nonetheless, the evidence presented here points to a number of actions for those working in and supporting the sector. The findings reinforce the observation that variegated and targeted support and policy schemes are needed when people in different areas (such as the performing arts compared with literature), are having such different experiences while also identifying the need to support better the financial stability of arts professionals (e.g., through guaranteed income or taxation schemes) [35]. This would mean that freelancers would not be forced to supplement their income with non-arts work if they did not want to, which could have ongoing impacts for training/rehearsal time, wellbeing, and potentially individuals’ longevity within their industries. Other workplace measures needed include protecting fair pay, contracts, and employment practices alongside a recognition of the distinctive challenges that early career and minority workers now face. Policy interventions should also be delivered alongside targeted support to address the ongoing mental health and wellbeing crisis in the sector, including information and initiatives that highlight the links between exercise, social networks, and wellbeing.

In practice, in the UK, initiatives that address some of these areas of action–including improvement of finances and health and wellbeing–are led by unions (such as the Musicians’ Union and the performing arts and entertainment trade union Equity), charities (such as the British Association for Performing Arts Medicine, BAPAM and Help Musicians), and networks (such as the Healthy Conservatoires Network). These organisations also provide support to individuals in the form of information and routes to support around finances (e.g., [88]), and health and wellbeing (such as routes to counselling). They also engage in advocacy to support arts professionals through government policy. The need for such advocacy has increased since the prioritisation of policies of growth within the creative industries since the financial crisis of 2008 which many regard as having been at the expense of working conditions and support of individual arts professionals [89]. Furthermore, the need for advocacy and new policies grows with every shift experienced by the sector, one notable example being the impact of existing and predicted growth of Artificial Intelligence in the arts [90]. Perhaps an element that is missing is the development of more collective “communities of care” [91] which can facilitate mentorship, professional networks, and focus on “collective rather than individual response to mitigating and managing this risk [associated with working in the creative industries], creating broader support structures in the absence of policy frameworks” ([91], p. 537). For individuals, there are a number of initiatives in place, but continued work is needed to ensure that professional artists are aware of those initiatives and feel they can engage in them, that there is an understanding of where professionals do have agency and control, and an acknowledgement of where they can have support when experiencing challenging circumstances beyond their control. More generally, both individual and collective practices are needed to address the issues identified in this study as the cultural sector develops.

Our data provide only a snapshot view of an evolving situation. Although the demographic and professional range reported here is wider than that reported in Phase 1 of our research, the article remains limited by the relatively small sample size in some professional areas and in some demographic groups. Nonetheless, methodologically speaking, the consistency of results across the two phases suggests that the models are replicable even under different circumstances–a year into the pandemic, with an expanded sample which included a broader range of arts professionals. The data so far indicate areas of stability and areas of change in arts professionals’ mental and social wellbeing as well as finances in the first year of the COVID-19 pandemic in the UK. But, the impact of pandemic restrictions has continued, and it is becoming increasingly intertwined with factors including Brexit and the cost of living crisis. In addition, at the time of these surveys, levels of COVID-19 infection were still relatively low. Rates of infection continue to fluctuate, which has implications for both arts professionals and their audiences in terms of, for example, long-COVID and for those who are clinically vulnerable. It is unclear how professionals in the arts will adapt as the situation evolves in the face of such economic uncertainty, in particular the balance of arts and non-arts work. In addition, there are ongoing impacts to early career professionals including lost opportunities, experiences, and financial hardship that have increased concerns about flight from the industry [11]. Whilst these results are specific to April–May 2021 (and COVID-19), they illustrate how the arts sector has experienced a time of crisis and has implications for how to prepare for future challenges and instability. Tracing patterns of long-term of mental and social wellbeing, work experiences, expectations, and satisfaction among arts professionals in the UK is therefore important to monitor and support the health of the cultural workforce.

## Conclusion

The results of Phase 2 of the *HEartS Professional Survey* suggest that the patterns of mental and social wellbeing along with their associated variables observed at the start of the pandemic remained broadly consistent a year on. More physical activity before lockdown was associated with higher wellbeing and social connectedness scores, and higher self-rated health scores were associated with higher wellbeing and lower depression scores. Similarly, increases in physical activity during lockdown, as well as older age, were associated with higher wellbeing and social connectedness scores and with lower depression and loneliness scores. Artists’ professional expectations of remaining in the arts were predicted by three contributors: greater proportion of income from the arts pre-pandemic, continued maintenance of skills, and greater proportion of freelance work. The results indicate possible strategies to support wellbeing and underline the importance of finances for expectations of remaining in arts professions. These support strategies include the need for variegated and targeted support and policy schemes, the need to support financial stability of arts professionals, and the need for targeted resources to address the ongoing mental health and wellbeing crisis in the sector, including information and initiatives that highlight the links between exercise, social networks, and wellbeing.

## Supporting information

S1 File *HEartS Professional* Survey II. (PDF)

S1 Table Sociodemographic, economic characteristics, and experience of COVID–19 of the sample. (PDF)

S2 Table General health and physical fitness characteristics. (PDF)

S3 Table Changes in work profiles and social meetings. (PDF)

S4 Table Mental health, wellbeing, and social outcome measures. (PDF)

S5 Table Multiple linear regressions modelling mental and social wellbeing. (PDF)

S6 Table Anticipation of future working in the arts. (PDF)

S7 Table Ordinal logistic regression modelling anticipated future in the arts. (PDF)
