# Supplementary material for: Modelling arts professionals’ wellbeing and career intentions within the context of COVID-19
Source: PLoS One. 2023 Oct 25;18(10):e0292722. doi: 10.1371/journal.pone.0292722 (PMC10599533; doi:10.1371/journal.pone.0292722)
Supplement: S1 Table — (PDF) [file pone.0292722.s002.pdf]

S1 TABLE | Sociodemographic, economic characteristics, and experience of COVID-19 of the sample, *HEartS Professional Survey II*, *N*=685.

	n	%
**Region (see Supplementary Figure 1, *HEartS Professional Survey II*, question 2.1)**		
Northern Scotland	4	1%
Southern Scotland	44	6%
North East	16	2%
North West	48	7%
Yorkshire and the Humber	51	7%
East Midlands	31	5%
West Midlands	43	6%
East of England	40	6%
South East	110	16%
South West	59	9%
London	211	31%
North Wales	3	0%
South Wales	22	3%
Northern Ireland	3	0%
	**Mean**	***SD***
**Age (see Supplementary Figure 1, *HEartS Professional Survey II*, question 2.2)**	37.84	13.35
**Categories**	***n***	**%**
18-25	96	14%
26-35	268	39%
36-45	134	20%
46-55	97	14%
56-65	67	9%
66-75	19	3%
76-94	4	1%
**Gender (see Supplementary Figure 1, *HEartS Professional Survey II*, question 2.3)**		
Men	233	34%
Women	436	64%
Non-binary/transgender	12	2%
Prefer not to say	2	0%

	n	%
**Ethnicity (see Supplementary Figure 1, *HEartS Professional Survey II*, question 2.4)**		
White British or Irish	545	80%
Any other White background	61	9%
Mixed ethnic backgrounds*	34	5%
Asian ethnic backgrounds*	24	4%
Black ethnic backgrounds*	11	2%
Any other ethnic background	10	2%

* *Ethnicity:* Mixed ethnic backgrounds includes White and Black Caribbean, White and Black African, White and Asian, and any other mixed ethnic background; Asian ethnic backgrounds includes Indian, Pakistani, Bangladeshi, Chinese, and any other Asian ethnic background; Black ethnic backgrounds includes Caribbean, African, and any other Black ethnic background.

**Professional specialisms (see Supplementary Figure 1, *HEartS Professional Survey II*, questions 2.5 and 4.1)**		
**Music or sound arts**	**199**	**29%**
Classical	112	56%
Dance (including electronic, house, techno)	33	17%
Jazz / blues / R'n'B	57	29%
Pop / rock	84	42%
Rap/Hiphop	30	15%
Folk/Country	35	18%
Musical Theatre	49	25%
Film / TV / theatre / radio / video games	9	5%
Other	38	19%
**Performing arts**	**218**	**32%**
Acting	97	45%
Dancing	40	18%
Circus arts / physical theatre/acrobatics/gymnastics	26	12%
Magic	5	2%
Musical theatre	75	34%
Puppetry	9	4%
Stand Up	17	8%
Other	92	42%

	n	%
**Visual arts**	**337**	**49%**
Drawing / illustration	77	24%
Ceramics	29	9%
Design	70	21%
Film/Video making/Photography	174	52%
Painting	58	17%
Printmaking	25	7%
Sculpture	43	13%
Cooking	7	2%
Floristry/Gardening	13	4%
Glass working/Jewellery Making	34	10%
Interior Design	11	3%
Metal/Wood working/Furniture Making	32	10%
Pottery	11	3%
Rug/Tapestry making	4	1%
Textiles	47	14%
Other	102	30%
**Literature**	**74**	**11%**
Non-fiction	38	51%
Fiction	48	65%
Film/TV Scripts	9	12%
Theatre	11	15%
Poetry	14	19%
Illustrations	10	14%
Broadcast Literature	4	5%
Journalism	18	24%
Translation	9	12%
Other	12	16%
**Education (see Supplementary Figure 1, *HEartS Professional Survey II*, question 2.8)**		
Secondary qualification (e.g. high school diploma)	104	15%
Tertiary / higher/ further qualification (e.g. bachelors degree)	399	58%
Advanced qualification (e.g. masters, PhD, DMA, DMus degree)	182	27%
**Living status (see Supplementary Figure 1, *HEartS Professional Survey II*, question 2.9)**		
On my own	89	13%
Residential care setting	1	0%
My spouse or partner	386	56%
Children	147	22%
Other family	114	17%
Friends or house share	85	12%
Other	7	1%

Would rather not say	3	0%
	n	**%**
**Household income (see Supplementary Figure 1, *HEartS Professional Survey II*, question 9.2)**		
Up to £5,199	23	3%
£5,200 and up to £10,399	37	5%
£10,400 and up to £15,599	43	6%
£15,600 and up to £20,799	51	7%
£20,800 and up to £25,999	70	10%
£26,000 and up to £31,199	67	10%
£31,200 and up to £36,399	46	7%
£36,400 and up to £41,599	57	8%
£41,600 and up to £46,799	34	5%
£46,800 and up to £51,999	47	7%
£52,000 and up to £75,999	87	13%
£76,000 and above	73	11%
Would rather not say	50	7%
	**Mean**	***SD***
**Percentage of household income contribution (%)**	53.1	30.83
**Percentage of contribution funded through arts work (%)**	63.44	37.00
**COVID-19 (see Supplementary Figure 1, *HEartS Professional Survey II*, question 3.1)**	***n***	**%**
Tested positive	35	5%
Have or previously had symptoms	91	13%
Not that know of	559	82%
**Physical activity: Medium (see Supplementary Figure 1, *HEartS Professional Survey II*, question 7.7)**		
I didn't do any sports or other energetic activities	126	18%
Online alone	89	13%
Online with others	44	6%
Offline alone	306	45%
Offline with others	120	18%

[1] For general health, we used one item from the Short Form 36 (SF-36) Health Survey (Ware and Gandek, 1998).

[2] Physical activity was measured using a scale from the Whitehall II Study, which measures frequency of engagement in activities that are mildly, moderately, and vigorously energetically taxing. Frequency is rated on a 4-point scale, from 0 *Hardly ever or never* to 3 *3 times a week or more* (Marmot and Brunner, 2005).
