# Supplementary material for: Modelling arts professionals’ wellbeing and career intentions within the context of COVID-19
Source: PLoS One. 2023 Oct 25;18(10):e0292722. doi: 10.1371/journal.pone.0292722 (PMC10599533; doi:10.1371/journal.pone.0292722)
Supplement: S2 Table — (PDF) [file pone.0292722.s003.pdf]

**S2 TABLE |** General health and physical fitness characteristics, *HEartS Professional Survey II*, *N*=685.

| | *n* | % |
|---|---|---|
| **Self-rated health[1] (see Supplementary Figure 1, *HEartS Professional Survey II*, question 7.3)** | | |
| Very good | 206 | 30% |
| Good | 288 | 42% |
| Fair | 157 | 23% |
| Bad | 29 | 4% |
| Very bad | 5 | 1% |
| **Ongoing (chronic) health issues (see Supplementary Figure 1, *HEartS Professional Survey II*, question 7.4)** | | |
| No | 477 | 70% |
| Yes | 186 | 27% |
| Would rather not say | 22 | 3% |
| Mental health problems | 37 | 20% |
| Chronic musculoskeletal problems | 4 | 2% |
| Cancer | 2 | 1% |
| Cardiovascular disease | 3 | 2% |
| Chronic respiratory disease | 11 | 6% |
| Chronic pain | 22 | 12% |
| Other | 45 | 24% |
| More than one health condition | 62 | 34% |

**Physical activity: Frequency[2] pre-COVID 19 (see Supplementary Figure 1, *HEartS Professional Survey II*, question 7.5)**

| | Mild | | Moderate | | Vigorous | | Total | |
|---|---|---|---|---|---|---|---|---|
| | *n* | % | *n* | % | *n* | % | *n* | % |
| Hardly ever or never | 32 | 5% | 92 | 13% | 274 | 40% | 398 | 19% |
| About once to 3 times a month | 43 | 6% | 128 | 19% | 131 | 19% | 302 | 15% |
| Once or twice a week | 166 | 24% | 249 | 36% | 154 | 23% | 569 | 28% |
| 3 times a week or more | 444 | 65% | 216 | 32% | 126 | 18% | 786 | 38% |

| | *n* | % |
|---|---|---|
| **Physical activity: Frequency post-COVID 19 (see Supplementary Figure 1, *HEartS Professional Survey II*, question 7.6)** | | |
| Much less often | 89 | 13% |
| Quite a lot less often | 99 | 15% |
| A little less often | 104 | 15% |
| No change | 186 | 27% |
| A little more often | 111 | 16% |
| Quite a lot more often | 70 | 10% |
| Much more often | 26 | 4% |

| | *n* | % |
|---|---|---|
| **Physical activity: Medium (see Supplementary Figure 1, *HEartS Professional Survey II*, question 7.7)** | | |
| I didn't do any sports or other energetic activities | 126 | 18% |
| Online alone | 89 | 13% |
| Online with others | 44 | 6% |
| Offline alone | 306 | 45% |
| Offline with others | 120 | 18% |

[1] For general health, we used one item from the Short Form 36 (SF-36) Health Survey (Ware and Gandek, 1998).
[2] Physical activity was measured using a scale from the Whitehall II Study, which measures frequency of engagement in activities that are mildly, moderately, and vigorously energetically taxing. Frequency is rated on a 4-point scale, from 0 *Hardly ever or never* to 3 *3 times a week or more* (Marmot and Brunner, 2005).
