# Supplementary material for: Modelling arts professionals’ wellbeing and career intentions within the context of COVID-19
Source: PLoS One. 2023 Oct 25;18(10):e0292722. doi: 10.1371/journal.pone.0292722 (PMC10599533; doi:10.1371/journal.pone.0292722)
Supplement: S3 Table — (PDF) [file pone.0292722.s004.pdf]

S3 TABLE | Changes in work profiles and social meetings, *HEartS Professional Survey II*, *N*=685.

3A. Time spent doing areas of work since the start of COVID-19
(see Supplementary Figure 1, *HEartS Professional Survey II*, question 4.4)

	Less time		No change		More time	
	n	**%**	***n***	**%**	***n***	**%**
Performing (*n*=192)	174	91%	5	3%	13	7%
Conducting / directing / producing (*n*=124)	81	65%	20	16%	23	19%
Teaching / coaching / workshop leading / mentoring (*n*=171)	98	57%	16	9%	57	33%
Managing / promoting (*n*=159)	84	53%	32	20%	43	27%
Composing / choreographing / designing / making / writing (*n*=311)	138	44%	62	20%	111	36%
Appraising / assessing / evaluating / reviewing (*n*=112)	42	38%	35	31%	35	31%
Researching (*n*=119)	34	29%	28	24%	57	48%
Curating / editing / presenting / technical supporting (*n*=213)	102	48%	50	24%	61	29%
Other (*n*=54)	24	45%	18	33%	12	22%
**Average %**		**52%**		**20%**		**28%**

3B. Medium (online or offline) and social context of work activities
(see Supplementary Figure 1, *HEartS Professional Survey II*, questions 4.5 and 4.6)

	Pre-COVID-19		Currently	
	n	**%**	***n***	**%**
**Performing (*n*=192)**				
Online alone	9	5%	60	31%
Online with others	12	6%	60	34%
Offline alone	19	10%	38	20%
Offline with others	152	79%	28	15%
**Conducting / directing / producing (*n*=124)**				
Online alone	9	7%	31	25%
Online with others	16	13%	48	39%
Offline alone	15	12%	26	21%
Offline with others	84	68%	19	15%
**Teaching / coaching / workshop leading / mentoring (*n*=171)**				
Online alone	8	5%	62	36%
Online with others	17	10%	92	54%
Offline alone	25	15%	8	5%
Offline with others	121	71%	9	5%

cont…

	Pre-COVID-19		Currently	
	n	%	*n*	%
**Managing / promoting (*n*=159)**				
Online alone	44	28%	78	49%
Online with others	32	20%	72	45%
Offline alone	11	7%	6	4%
Offline with others	72	45%	3	2%
**Composing / choreographing / designing / making / writing (*n*=311)**				
Online alone	52	17%	88	28%
Online with others	26	8%	63	20%
Offline alone	150	48%	148	48%
Offline with others	83	27%	12	4%
**Appraising / assessing / evaluating / reviewing (*n*=112)**				
Online alone	27	24%	55	49%
Online with others	17	15%	35	31%
Offline alone	22	20%	19	18%
Offline with others	46	41%	3	3%
**Researching (*n*=119)**				
Online alone	62	52%	80	67%
Online with others	17	14%	23	19%
Offline alone	19	16%	15	13%
Offline with others	21	18%	1	1%
**Curating / editing / presenting / technical supporting (*n*=213)**				
Online alone	44	21%	75	35%
Online with others	39	18%	87	41%
Offline alone	39	18%	38	18%
Offline with others	91	43%	13	6%
**Other (*n*=54)**				
Online alone	7	13%	17	32%
Online with others	6	11%	24	44%
Offline alone	5	9%	7	13%
Offline with others	36	67%	6	11%

3C. Skill maintenance and development
(see Supplementary Figure 1, *HEartS Professional Survey II*, questions 4.7 and 4.8)

Learning / practising / preparing / reflecting…	individually		with others online		with other in person		overall	
	n	**%**	***n***	**%**	***n***	**%**	**mean *n***	**mean %**
I haven't done it	32	5%	89	13%	225	33%	115	17%
Less*	239	35%	129	19%	356	52%	241	35%
No change	125	18%	111	16%	70	10%	102	15%
More*	289	42%	356	52%	34	5%	226	33%

3D. Changes in socializing in the past month
(see Supplementary Figure 1, *HEartS Professional Survey II*, questions 3.3 and 3.4)

	In person		Electronically	
	n	**%**	***n***	**%**
Fewer people*	583	85%	77	11%
No change	52	8%	169	25%
More people*	50	7%	439	64%

3E. Changes in finance
(see Supplementary Figure 1, *HEartS Professional Survey II*, question 9.6)

	n	**%**
Increase*	150	23%
No change	324	49%
Decrease*	189	28%

3F. Financial hardship
(see Supplementary Figure 1, *HEartS Professional Survey II*, question 5.7)

	n	**%**
Yes	405	59%
Yes, a lot	128	19%
Yes, a little	277	40%
No	280	41%

3G. Changes in loneliness and anxiety
(see Supplementary Figure 1, *HEartS Professional Survey II*, questions 3.6 and 3.7)

	Lonely		Anxious	
	n	**%**	***n***	**%**
More*	436	64%	487	71%
No change	188	27%	155	23%
Less*	61	9%	43	6%

* *For Tables 3A, 3C, and 3E:* 'More' includes 'Much more, quite a lot more, and a little more'; 'Less' includes 'Much less, quite a lot less, and a little less'. *For Table 3D:* 'Fewer people' includes 'Substantially fewer people, far fewer people, and fewer people'; 'More people' includes 'Substantially more people, far more people, and more people'.

3H. Sources of support
(see Supplementary Figure 1, *HEartS Professional Survey II*, questions 5.9, 5.10, 5.12, 5.13)

	n	%
**Financial support**	**405**	
Yes	232	57%
No	173	43%
**If yes, where?**	**232**	
Colleagues	72	31%
Charities (arts-specific)	62	27%
Charities (general)	28	12%
Educational institutions	24	10%
Employers	39	17%
Family / friends	154	66%
Finance professionals (arts-specific) / advisors / banks / finance helplines	21	9%
Finance professionals (general) / advisors / banks / finance helplines	45	19%
Government-based agencies	107	46%
Insurers	3	1%
Teachers / coaches / mentors	15	7%
Trade unions	30	13%
Other	9	4%

	n	%
**Health and wellbeing support**	**405**	
Yes	228	56%
No	177	44%
**If yes, where?**	**228**	
Colleagues	58	25%
Charities (arts-specific)	20	9%
Charities (general)	29	13%
Educational institutions	12	5%
Employers	9	4%
Family / friends	161	71%
Health professionals (arts-specific) / advisors / hospitals / helplines	19	8%
Health professionals (general) / advisors / hospitals / helplines	101	44%
Government-based agencies	17	8%
Insurers	0	0%
Teachers / coaches / mentors	14	6%
Trade unions	11	12%
Other	16	7%
