# Supplementary material for: Modelling arts professionals’ wellbeing and career intentions within the context of COVID-19
Source: PLoS One. 2023 Oct 25;18(10):e0292722. doi: 10.1371/journal.pone.0292722 (PMC10599533; doi:10.1371/journal.pone.0292722)
Supplement: S4 Table — (PDF) [file pone.0292722.s005.pdf]

Spiro N, Shaughnessy C, Waddell G, Perkins R, Campbell A, Williamon A (2023), Modelling arts professionals' wellbeing and career intentions within the context of COVID-19, *PLOS One*.

S4 TABLE | Mental health, wellbeing, and social outcome measures, *HEartS Professional Survey II*, *N*=685.

**Mental Health Continuum – Short Form (MHC-SF) 14-item scale[1] (see Supplementary Figure 1, *HEartS Professional Survey II*, question 7.1)**		
Score (0–70): Mean, *SD*	35.3	13.49
Categorical variable: *n*, %		
Languishing	129	19%
Moderate wellbeing	382	56%
Flourishing	174	25%

**Centre for Epidemiologic Studies Depression Scale (CES-D) Short Form 8-item scale[2] (see Supplementary Figure 1, *HEartS Professional Survey II*, question 7.2)**		
Score (0–8): Mean, *SD*	4.27	2.54
Number of depression cases (≥ 3 score): *n*, %	498	73%

**Social Connectedness Scale-Revised (SCS-R) 15-item scale[3] (see Supplementary Figure 1, *HEartS Professional Survey II*, question 8.1)**		
Score (0–75): Mean, *SD*	39.46	15.38

**Three-Item Loneliness Scale (UCLA)[4] (see Supplementary Figure 1, *HEartS Professional Survey II*, question 8.2)**		
Score (3-9): Mean, *SD*	5.56	1.76
Number of lonely cases (≥6 score): *n*, %	362	53%

[1] Higher scores indicate better wellbeing (Keyes 2002, 2005).
[2] Scores of 3 or more indicate depression (Karim et al., 2015).
[3] Higher scores indicate better levels of social connectedness (Lee et al., 2008).
[4] Scores of 6 or higher indicate loneliness (Steptoe et al., 2013).
**p* < .001.

References

Karim, J., Weisz, R., Bibi, Z., & ur Rehman, S. (2015). Validation of the eight-item Center for Epidemiologic Studies Depression Scale (CES-D) among older adults. *Current Psychology*, *34*(4), 681–692. https://doi.org/10.1007/s12144-014-9281-y

Keyes CLM (2002), The mental health continuum: From languishing to flourishing in life, *J Health Soc Behav* 43, doi: 10.2307/3090197

Keyes CLM (2005), Mental illness and/or mental health? Investigating axioms of the complete state model of health, *J Consult Clin Psychol* 73, doi: 10.1037/0022-006X.73.3.539

Lee RM, Dean BL, and Jung K-R (2008), Social connectedness, extraversion, and subjective well-being: Testing a mediation model, *Pers Individ Dif* 45, doi: 10.1016/j.paid.2008.05.017

Steptoe A, Shankar A, Demakakos P, and Wardle J. (2013), Social isolation, loneliness, and all-cause mortality in older men and women. *Proc Natl Acad Sc*i 110: 5797–801, doi:10.1073/pnas.1219686110
