# Supplementary material for: Modelling arts professionals’ wellbeing and career intentions within the context of COVID-19
Source: PLoS One. 2023 Oct 25;18(10):e0292722. doi: 10.1371/journal.pone.0292722 (PMC10599533; doi:10.1371/journal.pone.0292722)
Supplement: S5 Table — (PDF) [file pone.0292722.s006.pdf]

Spiro N, Shaughnessy C, Waddell G, Perkins R, Campbell A, Williamon A (2023), Modelling arts professionals' wellbeing and career intentions within the context of COVID-19, *PLOS One*.

S5 TABLE | Multiple linear regressions modelling mental and social wellbeing

Variables used in the multiple linear regressions and their descriptors

	Descriptor
COVID-19-Specific	
Lockdown exercise	Continuous: Changes in physical activity during lockdown (-3 to 3).<br>Much less physical activity = -3, Quite a bit less physical activity = -2, Little less physical activity = -1, No change = 0, A little more physical activity = 1, Quite a bit more physical activity = 2, Much more physical activity = 3.
Socializing change	Continuous: Sum of self-reported change in socializing with others in-person and online.
Financial hardship	Binary: Yes, a little *or* Yes, a lot = 1, No = 0
Demographics	
Gender	Binary: Male = 1, Female *or* Other = 0,
Ethnicity	Binary: White = 1, Non-white = 0
Age	Continuous: Participants' ages were represented in whole numbers
Health	Very good = 2, Good = 1, Fair = 0, Bad = -1, Very bad = -2
Pre-COVID-19 exercise	Continuous: frequency of exercise pre-COVID (0-12)<br>Composite calculated variable comprising sum of:<br>Mild exercise: Never/hardly ever = 0, About once to 3 times a month = 1, once or twice a week = 2, 3 times a week or more = 3<br>Moderate exercise: Never/hardly ever = 0, About once to 3 times a month = 2, once or twice a week = 3, 3 times a week or more = 4<br>Vigorous exercise: Never/hardly ever = 0, About once to 3 times a month = 3, once or twice a week = 4, 3 times a week or more = 5
Living alone	Binary: Living alone = 1, Other living configurations = 0
Educational attainment	Binary: Advanced degree or equivalent (e.g. masters, doctorate)= 1, Other qualifications = 0
Household income	Continuous
% Freelance	Continuous, Self-report
% Contribution to household income	Continuous, Self-report
% Contribution of income from arts	Continuous, Self-report
Arts area	Categorical

5A. Multiple linear regression modelling wellbeing, *N* = 685

	Model 1						Model 2					
	B	*SE B*	β	*p*	95% CI		*B*	*SE B*	β	*p*	95% CI	
(Constant)	38.68	0.95		<.001	36.82	40.55	24.43	3.08		<.001	18.37	30.49
COVID-19-Specific												
**Lockdown exercise**	**1.01**	**0.31**	**0.12**	**.002**	**0.36**	**1.66**	**0.96**	**0.32**	**0.12**	**.003**	**0.32**	**1.59**
**Socializing change**	**0.99**	**0.34**	**0.12**	**.003**	**0.33**	**1.65**	**0.89**	**0.33**	**0.11**	**.007**	**0.24**	**1.53**
Financial hardship	**-3.23**	**1.11**	**-0.24**	**.004**	**-5.40**	**-1.05**	-1.76	1.15	-0.13	.126	-4.01	0.49
Demographics												
Gender							1.14	1.12	0.08	.312	-1.07	3.34
Ethnicity							-0.51	1.65	-0.04	.754	-3.77	2.73
**Age**							**0.12**	**0.04**	**0.12**	**.007**	**0.03**	**0.20**
**Health**							**3.31**	**0.63**	**0.21**	**<.001**	**2.05**	**4.55**
**Pre-COVID-19 exercise**							**0.42**	**0.17**	**0.10**	**.012**	**0.09**	**0.76**
Living alone							-0.72	1.84	-0.05	.698	-4.33	2.90
Ed. attainment							0.55	1.23	0.04	.655	-1.87	2.97
Household income							0.18	0.18	0.05	.314	-0.17	0.54
% Freelance							0.00	0.01	0.00	.983	-0.03	0.03
% Cont. income							0.01	0.02	0.02	.656	-0.03	0.05
% Cont. from art							0.02	0.02	0.04	.322	-0.02	0.05
Arts area							-0.92	1.12	-0.07	.412	-3.12	1.30
R^2	.046						.137					
Adjusted R^2	.041						.114					
ΔR^2	.046						.091					
F	9.40***						6.08***					
ΔF							5.06***					

Abbreviations: B, unstandardized beta; SEB, standard error of B; β, standardized beta, CI, confidence interval.
Note: *N*=685, **p* < .05, ***p* < .01, ****p* < .001, Gender = male, Ethnicity = white. Starting model: df (3, 587), VIF<1.01, Tol>0.99. Final model: *df* (15, 575), VIF <1.80, Tol>0.55. Durbin-Watson = 1.97.

5B. Multiple linear regression modelling depression, *N* = 685

	**Model 1**						**Model 2**					
	B	*SE B*	β	*p*	95% CI		*B*	*SE B*	β	*p*	95% CI	
(Constant)	3.48	0.18		<.001	3.12	3.83	5.97	0.58		<.001	4.74	7.01
COVID-19-Specific												
**Lockdown exercise**	**-0.19**	**0.18**	**-0.12**	**.002**	**-0.31**	**-0.07**	**-0.17**	**0.06**	**-0.11**	**.005**	**-0.29**	**-0.05**
Socializing change	**-0.13**	**0.06**	**-0.08**	**.038**	**-0.26**	**0.01**	-0.12	0.06	0.08	.051	-0.24	0.00
**Financial hardship**	0.92	0.21	0.36	<.001	0.51	1.33	**0.63**	**0.21**	**0.25**	**.003**	**0.21**	**1.06**
Demographics											.	
**Gender**							**-0.51**	**0.21**	**-0.20**	**.015**	**-0.92**	**-0.10**
Ethnicity							-0.02	0.31	0.01	.948	-0.63	0.59
**Age**							**-0.03**	**0.01**	**-0.15**	**<.001**	**-0.05**	**-0.01**
**Health**							**-0.76**	**0.12**	**-0.26**	**<.001**	**-0.99**	**-0.52**
Pre-COVID-19 exercise							-0.02	0.03	-0.02	.566	-0.08	0.04
Living alone							0.23	0.34	0.09	.513	-0.45	0.90
Ed. attainment							-0.01	0.23	-0.00	.963	-0.46	0.44
% Freelance							-0.01	0.03	-0.01	.876	-0.07	0.06
Household income							-0.00	0.00	0.03	.512	-0.01	0.00
% Cont. income							0.00	0.00	0.02	.761	-0.01	0.01
% Cont. from art							-0.00	0.00	-0.05	.297	-0.01	0.00
Arts area							0.41	0.21	0.16	.051	0.00	0.82
R^2	.055						.160					
Adjusted R^2	.050						.139					
ΔR^2	.055						.105					
F	11.41***						7.35***					
ΔF							6.03***					

Abbreviations: B, unstandardized beta; SEB, standard error of B; β, standardized beta; CI, confidence interval.
Note: *N*=685, *p < .05, **p < .01, ***p < .001, Gender = male, Ethnicity = white. Starting model: df (3, 587), VIF<1.01, Tol>0.99. Final model: *df* (15, 575), VIF <1.80, Tol>0.55. Durbin-Watson = 1.91.

5C. Multiple linear regression modelling social connectedness, *N* = 685

	Model 1						Model 2					
	B	*SE B*	β	*p*	95% CI		*B*	*SE B*	β	*p*	95% CI	
(Constant)	42.85	1.09		<.001	45.85	50.90	25.47	3.55		<.001	18.52	32.44
COVID-19-Specific												
**Lockdown exercise**	**1.06**	**0.38**	**0.11**	**.005**	**0.27**	**1.37**	**0.95**	**0.36**	**0.10**	**.010**	**0.23**	**1.68**
**Socializing change**	**1.48**	**0.38**	**0.16**	**<.001**	**-0.43**	**0.89**	**1.45**	**0.38**	**0.15**	**<.001**	**0.70**	**2.19**
Financial hardship	-2.49	1.26	-0.16	.051	-1.28	0.03	-1.44	1.31	-0.09	.275	-4.03	1.15
Demographics												
Gender							0.57	1.29	-0.04	.660	-1.97	3.10
Ethnicity							0.11	1.90	0.01	.953	-3.62	3.85
**Age**							**0.13**	**0.05**	**0.11**	**.009**	**0.03**	**0.23**
**Health**							**3.23**	**0.73**	**0.18**	**<.001**	**1.79**	**4.66**
**Pre-COVID-19 exercise**							**0.41**	**0.19**	**0.09**	**.033**	**0.03**	**0.80**
Living alone							-3.39	2.12	-0.22	.110	-7.55	0.77
Ed. attainment							0.02	1.41	0.00	.985	-2.75	2.80
Household income							0.30	0.21	0.07	.148	-0.11	0.71
% Freelance							-0.00	0.02	-0.00	.952	-0.03	0.03
% Cont. income							0.03	0.03	0.06	.248	-0.02	0.08
% Cont. from art							0.01	0.02	0.03	.442	-0.02	0.05
Arts area							1.74	1.29	0.11	.174	-0.78	4.28
R^2	.046						.130					
Adjusted R^2	.041						.108					
ΔR^2	.046						.084					
F	9.47**						5.75***					
ΔF							4.64***					

Abbreviations: B, unstandardized beta; SEB, standard error of B; β, standardized beta; CI, confidence interval.
Note: *N*=685, **p* < .05, ***p* < .01, ****p* < .001, Gender = male, Ethnicity = white. Starting model: df (3, 587), VIF<1.01, Tol>0.99. Final model: *df* (15, 575), VIF <1.80, Tol>0.55. Durbin-Watson = 1.90.

5D. Multiple linear regression modelling loneliness, *N* = 685

	Model 1						Model 2					
	B	*SE B*	B	*p*	95% CI		*B*	*SE B*	β	*p*	95% CI	
(Constant)	5.04	0.12		<.001	4.47	5.26	5.87	0.40		<.001	5.08	6.67
COVID-19-Specific												
**Lockdown exercise**	**-0.15**	**0.04**	**-0.14**	**<.001**	**-0.21**	**-0.04**	**-0.13**	**0.04**	**-0.13**	**.001**	**-0.22**	**-0.05**
Socializing change	-0.07	0.04	-0.07	.101	-0.10	0.10	-0.08	0.04	-0.07	.071	-0.16	0.01
**Financial hardship**	**0.61**	**0.14**	**0.35**	**<.001**	**-0.03**	**0.23**	**0.51**	**0.15**	**0.29**	**<.001**	**0.21**	**0.80**
Demographics												
Gender							-0.26	0.15	-0.15	.075	-0.55	0.03
Ethnicity							-0.28	0.22	-0.16	.202	-0.15	0.70
**Age**							**-0.03**	**0.01**	**-0.21**	**<.001**	**-0.04**	**-0.02**
**Health**							**-0.28**	**0.08**	**-0.14**	**<.001**	**-0.45**	**-0.12**
Pre-COVID-19 exercise							-0.02	0.02	-0.03	.487	-0.06	0.03
Living alone							0.42	0.24	0.24	.079	-0.05	0.89
Ed. attainment							0.26	0.16	0.15	.101	-0.05	0.58
Household income							0.01	0.02	-0.01	.782	-0.04	0.05
% Freelance							0.00	0.00	0.04	.341	-0.00	0.01
% cont. income							0.00	0.00	0.04	.462	-0.00	0.01
% cont. from art							0.00	0.00	-0.02	.726	-0.00	0.00
Arts area							0.11	0.15	0.06	.455	-0.18	0.40
R^2	.054						.136					
Adjusted R^2	.050						.114					
ΔR^2	.054						.092					
F	11.25**						6.08***					
ΔF							5.06***					

Abbreviations: B, unstandardized beta; SEB, standard error of B; β, standardized beta; CI, confidence interval.
Note: *N*=353, $*p < .05$, $**p < .01$, $***p < .001$, Gender = male, Ethnicity = white. Starting model: df (4, 352), VIF<1.03, Tol>0.98. Final model: *df* (16, 352), VIF <1.76, Tol>0.57. Durbin-Watson = 1.99.
