# Supplementary material for: Modelling arts professionals’ wellbeing and career intentions within the context of COVID-19
Source: PLoS One. 2023 Oct 25;18(10):e0292722. doi: 10.1371/journal.pone.0292722 (PMC10599533; doi:10.1371/journal.pone.0292722)
Supplement: S6 Table — (PDF) [file pone.0292722.s007.pdf]

S6 TABLE | Anticipation of future working in the arts, HEartS Professional Survey II, *N* = 685

	n	**%**
**Anticipation of future in the arts (see Supplementary Figure 1, *HEartS Professional Survey II*, question 5.7a)**	**685**	
Yes, purely in the arts	281	41%
Yes, purely in the arts but not at the moment	50	7%
Yes, in the arts alongside non-arts work	194	28%
Yes, in the arts alongside non-arts work, but not at the moment	39	6%
Maybe, in the arts alongside non-arts work	63	9%
Maybe, in the arts alongside non-arts work, but not at the moment	36	5%
No	21	3%
