# Supplementary material for: Modelling arts professionals’ wellbeing and career intentions within the context of COVID-19
Source: PLoS One. 2023 Oct 25;18(10):e0292722. doi: 10.1371/journal.pone.0292722 (PMC10599533; doi:10.1371/journal.pone.0292722)
Supplement: S7 Table — (PDF) [file pone.0292722.s008.pdf]

S7 TABLE | Ordinal logistic regression modelling anticipated future in the arts, *N* = 685

	**Model 1**						**Model 2**					
	B	*SE B*	OR	*p*	95% CI (OR)		*B*	*SE B*	OR	*p*	95% CI (OR)	
Health and wellbeing												
Depression	-0.00	0.05	1.00	.950	0.90	1.10	0.02	0.05	1.02	.724	0.92	1.13
Loneliness	0.01	0.07	1.01	.905	0.88	1.16	-0.01	0.07	0.99	.931	0.86	1.15
Social connectedness	0.01	0.01	1.01	.357	0.99	1.03	0.01	0.01	1.01	.235	0.99	1.03
**Wellbeing**	**0.02**	**0.01**	**1.02**	**.029**	**1.00**	**1.04**	0.02	0.01	1.02	.121	1.00	1.04
Pre-COVID-19 exercise	-0.05	0.03	0.95	.085	0.90	1.01	-0.05	0.03	0.95	.072	0.89	1.00
**Connection to others arts**	**0.16**	**0.07**	**1.18**	**.027**	**1.02**	**1.36**	0.13	0.08	1.13	.093	0.98	1.32
Socializing change	-0.01	0.06	0.99	.901	0.89	1.11	-0.06	0.06	0.94	.309	0.83	1.06
Long-COVID Impact	0.29	0.32	1.33	.367	0.73	2.57	0.10	0.33	1.11	.757	0.59	2.20
Health	0.05	0.06	1.05	.664	0.84	1.31	0.04	0.12	1.05	.707	0.83	1.32
Financial												
Financial hardship							-0.31	0.21	0.73	.141	0.48	1.11
Household income							-0.03	0.03	0.97	.382	0.91	1.03
**% Freelance**							**0.01**	**0.00**	**1.01**	**.020**	**1.00**	**1.01**
% Cont. income							0.00	0.00	1.00	.465	1.00	1.01
**% Cont. from art**							**0.01**	**0.00**	**1.01**	**.004**	**1.00**	**1.01**
**Current skill maint.**							**0.27**	**0.08**	**1.32**	**<.001**	**1.13**	**1.53**
Future skill maint.							0.13	0.08	1.14	.092	0.98	1.33
Fund recuperation							-0.01	0.07	0.99	.833	0.86	1.13
R^2_{McF}	.031						.070					
ΔR^2_{McF}	.031						.039					
χ^2	33.06***						74.62***					
$\Delta\chi^2$							41.56***					

Abbreviations: B, unstandardized beta; SEB, standard error of B; OR, odds ratio, CI (OR), confidence interval of the odds ratio
Note: *N*=685, **p* < .05, ***p* < .01, ****p* < .001, Gender = male, Ethnicity = white. Starting model: AIC = 1056, BIC = 1109. Final model: AIC = 1030, BIC = 1118.
